# Infectious Bronchitis Virus (IBV) in Vaccinated and Non-Vaccinated Broilers in Brazil: Surveillance and Persistence of Vaccine Viruses

**DOI:** 10.3390/microorganisms13030521

**Published:** 2025-02-27

**Authors:** Gleidson Biasi Carvalho Salles, Giulia Von Tönnemann Pilati, Beatriz Pereira Savi, Mariane Dahmer, Eduardo Correa Muniz, Josias Rodrigo Vogt, Antonio José de Lima Neto, Gislaine Fongaro

**Affiliations:** 1 Laboratory of Applied Virology, Department of Microbiology, Immunology and Parasitology, Federal University of Santa Catarina, Florianópolis 88040-900, SC, Brazilgiuliavpilati@gmail.com (G.V.T.P.); beasavis2@gmail.com (B.P.S.); marianedahmer@gmail.com (M.D.); 2 Zoetis Industry of Veterinary Products LTDA, São Paulo 04709-111, SP, Brazil; eduardo.muniz@zoetis.com (E.C.M.); josias.vogt@zoetis.com (J.R.V.);

**Keywords:** infectious bronchitis virus, vaccine viral escapes, vaccination, emerging variant strains, poultry

## Abstract

Infectious bronchitis virus (IBV) poses a significant threat to poultry worldwide, necessitating robust surveillance and vaccination strategies. This study aimed to conduct IBV surveillance in Brazil, assess potential vaccine viral escapes, and evaluate vaccine persistence in vaccinated broilers. A total of 1000 tracheal swabs from 100 flocks across six states were analyzed using RT-PCR. The results showed that 91% of the flocks tested positive for IBV. The detected strains included GI-1, GI-11, and GI-23. Notably, 90% of batches received vaccines containing either GI-1 or GI-11 lineages. The study revealed vaccine persistence in 67 samples between days 16 and 32 post-vaccination. In contrast, unvaccinated batches had a high prevalence of IBV GI-11 strains (70%). These findings highlight widespread IBV circulation in Brazil with persistent viral presence in vaccinated birds and wild viruses in unvaccinated ones. Collectively, the data reveal a widespread presence of IBV in Brazil, characterized by prolonged viral persistence in vaccinated animals and the occurrence of wild viruses in both unvaccinated birds and those vaccinated against specific strains. It can be concluded from this study that there was a widespread occurrence of IBV in Brazil, providing long viral persistence in vaccinated animals, as well as the occurrence of wild virus in unvaccinated birds or birds vaccinated against individual strains.

## 1. Introduction

The historical transformation in global poultry farming has brought about numerous challenges, including the control of diseases, especially respiratory diseases in birds [1]. The concentration of poultry in specific regions has significantly increased sanitary risks, leading to the introduction of many diseases in various parts of the world. In this context, infectious bronchitis virus (IBV) was first reported in the 1930s in the United States, with the identification of the mass serotype (GI-1) [2]. Within a few years, it was identified in various regions across the globe, characterizing the virus as omnipresent in the worldwide poultry industry [3].

Infectious bronchitis is a highly contagious viral disease that affects chickens (*Gallus gallus domesticus*) as well as other domestic and wild birds, including partridges, geese, pigeons, guinea fowl, mallards, ducks, and peacocks [4]. The etiological agent of the disease is the infectious bronchitis virus (IBV), which belongs to the *Coronaviridae* family, order Nidovirales. The Orthocoronavirinae subfamily is divided into four genera: *Alphacoronavirus*, *Betacoronavirus*, *Gammacoronavirus*, and *Deltacoronavirus*, with IBV being part of the *Gammacoronavirus* genus [5,6,7,8].

The genome consists of a single-stranded positive-sense RNA with a length of 27–28 kb that encodes both structural and non-structural proteins (NSPs). The proximal two-thirds of the genome (5′ end) encodes two overlapping open reading frames (ORFs), ORF1a and ORF1b, which together code for 15 non-structural proteins primarily involved in RNA replication and transcription [4,9]. The genomic organization follows the pattern: 5′ untranslated region (UTR)—leader—ORF1a/1ab—S—3a—3b—E—M—5a—5b—N—3′ UTR. Consequently, the remaining one-third of the genome encodes the structural proteins, including the spike glycoprotein (S), envelope protein (E), membrane protein (M), and nucleocapsid protein (N) [10]. Based on complete sequences obtained from the S1 gene, it is possible to define six genotypes comprising 32 distinct lineages (GI-1~GI-27, GII-1, GII-2, GIV-1, GV-1, and GVI-1) [11,12].

The disease is primarily characterized by upper respiratory symptoms in birds, but it is also common to affect other organs such as the kidneys and the reproductive tract. This can lead to issues related to airsacculitis, proventriculitis, nephritis, zootechnical losses, poor egg quality, high morbidity, and mortality [2,13].

One of the primary methods for controlling IBV involves implementing a combination of effective production practices and stringent biosecurity measures on poultry farms. Additionally, the use of live (replicating) or inactivated (non-replicating) vaccines has proven to be highly effective in managing this disease. However, the extensive genetic diversity and high mutation rate of IBV leads to the emergence of numerous distinct viral lineages, which diminishes the protective efficacy of vaccines. Consequently, careful selection and administration of vaccines tailored to the prevalent strains in specific geographic areas are crucial for achieving optimal protection against this virus [2].

The genetic evolution of IBV involves two primary mechanisms. The first mechanism entails the accumulation of mutations over time, driven by simple evolutionary processes or pressures exerted by vaccines, leading to genetic differentiation [14]. This process occurs due to errors made by the polymerase enzyme during viral RNA replication. As these mistakes happen rapidly and are not efficiently corrected, they result in the swift evolution of the virus [15]. When such errors confer a selective advantage on the virus, they give rise to new “genetic variants” that can emerge as disease-causing strains in birds [16]. The second mechanism driving genetic changes in IBV is recombination, which occurs when two different IBV strains infect the same cell simultaneously [16]. During replication, the polymerase enzyme switches between the genomes of these two viruses, creating a new viral genome that combines elements from both parent viruses. This process can lead to rapid and significant alterations in the viral genome composition [17]. Although recombination can result in substantial genetic changes, it often requires compatibility among specific genes involved in pathogenicity and immunogenicity for a new disease-causing virus to emerge. Recombination is not only a key mechanism for generating genetic and antigenic diversity but also plays a crucial role in shaping IBV evolution by facilitating adaptations that may lead to new strains or serotypes

This constant Darwinian evolution of IBV results in the emergence of new virus types [18]. Some types of IBV vaccines are particularly good at inducing antibodies that will cross-react with more distant virus types, providing broad protection, but each case is different and should be experimentally tested in chickens [19].

The substantial antigenic variability of IBV has led to the development of various vaccines worldwide, primarily live-attenuated and killed formulations based on local serotypes. Using two genetically distinct vaccines can provide broad, though incomplete, protection against heterologous viruses. Epidemiological surveillance can help characterize which genotype or lineage is present in a particular region, evaluate possible vaccine viral escapes, and identify new wild strains of IBV. When circulating IBV strains acquire mutations that allow them to evade immunity induced by existing vaccines, this can lead to reduced vaccine efficacy and an increased number of outbreaks, even in vaccinated populations [2,12,20,21,22].

In Brazil, IBV has been documented since the 1950s, revealing a diverse array of genotypes and lineages identified over the decades. Initially, the GI-1 (mass-type) was among the first strains to be detected [23]. The emergence of molecular biology techniques in the 1990s greatly improved our comprehension of IBV’s genetic diversity within Brazil. As of 2021, only two vaccine serotypes have received official approval for use: GI-1 and GI-11.

In 2022, a new lineage, GI-23 (Variant-2), was identified in Brazil, and a matching vaccine was immediately introduced [24,25]. The IBV belonging to the GI-23 lineage, was first described in Israel in 2004 and was responsible for significant lesions in the respiratory and nephropathogenic systems in birds. In 2016, the identification of the same strain in Europe was reported. Despite the use of inactivated and live-attenuated vaccines to control IBV in Brazil, outbreaks are frequent due to intensive breeding systems [12,26]. 

Therefore, this study aimed to conduct surveillance of IBV in Brazil, focusing on identifying vaccine viral escapes, and assessing the persistence of vaccine viruses in vaccinated broiler flocks. This objective aligns with addressing the challenges posed by IBV’s high genetic variability and its implications for vaccine efficacy.

## 2. Materials and Methods

### 2.1. Sampling

**Figure 1:**
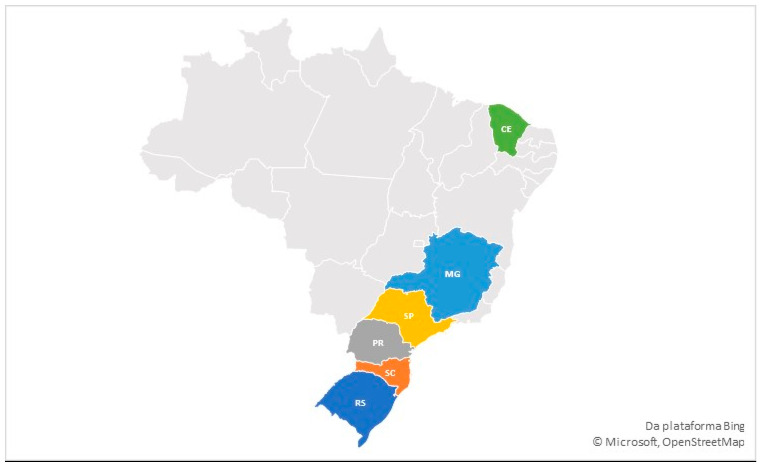
Map of Brazil, highlighting the South, Southeast and Northeast Regions and the Brazilian states sampled in the present study, being Rio Grande do Sul (RS), Santa Catarina (SC), Paraná (PR), São Paulo (SP), Minas Gerais (MG) and Ceará (CE).

A total of 100 broiler chicken batches (*Gallus gallus domesticus*) were collected and assessed. Figure 1 shows the collection regions and areas. The samples were sourced from various states in Brazil, gathered between August and December 2021. The number of samples collected from each state matched the proportional poultry production of that region. Ten chickens were selected from each batch, culminating in 1000 chickens from the following areas: south (Paraná—30 batches, Santa Catarina—15 batches, Rio Grande do Sul—15 batches), southeast (São Paulo—10 batches, Minas Gerais—10 batches), and northeast (Ceará—20 batches). These regions together account for 80% of Brazil’s broiler chicken production (ABPA, 2024 [27]). 

Sterilized swabs were used for collecting the tracheal biological material of 1000 chickens to detect IBV in the samples. All collection was carried out from dead animals, donated by the producing farms, and submitted to inspection aged between 13 and 32 days. The batches were selected according to their history of having presented some respiratory disease with unidentified pathogens, including sneezing and respiratory secretions. The Table 1 show the vaccination program of the batches.

All biological samples evaluated here were donated by farms that carry out routine inspections, eliminating the need for an ethics committee as they are leftover biological samples collected by routine health surveillance services—Consultation with the Ethics Committee on the Use of Animals (CEUA No. 4434190521/Federal University of Santa Catarina).

### 2.2. Extraction of Genetic Material and Molecular Detection of IBV

Tracheal swabs were eluted in PBS (1X), and total RNA extraction from the samples was performed using the RNeasy^®^ Mini kit (QIAGEN, Hilden, Germany) following the manufacturer’s instructions. The RNA quality and quantity was evaluated using the NanoVue™ spectrophotometer (Boston, MA, USA) and stored at −20 °C.

Reverse transcription followed by polymerase chain reaction (RT-PCR) was carried out to determine the presence of IBV, to the hyper-variable target region of the S1 gene of IBV. Reagents used included the High-Capacity cDNA Reverse Transcription Kit (Applied Biosystems, Waltham, MA, USA) and Platinum^TM^ Taq DNA Polymerase (Thermo Fisher Scientific, Waltham, MA, USA), generating amplicons of 392 base pairs (bp) corresponding to the hyper-variable target region of the S1 gene of IBV [28]. A second amplification reaction was performed using a nested PCR assay targeting the hypervariable region 3 of spike gene (393 bp) sequences on cDNA of all positive samples in real-time PCR step. The SX1 (5′-CACCTAGAGGTTTGYTWGCATG-3′) and SX2 (5′-TCCACCTCTATAAACACCYTTAC-3′) primer set were used in first reaction while SX3 (5′-TAATACTGGYAATTTTTCAGATGG-3′) and SX4 (5′ AATACAGATTGCTTACAACCACC-3′) primers were employed in the second round [29]. The amplicons were subjected to horizontal agarose gel electrophoresis at 1%, using GelRed as a DNA intercalating agent.

The IBV positive samples were subjected to genotyping, based on the analysis of complete or partial S1 sequence, using the Sanger method to obtain phylogenetic information and confirm the viral lineage and strain [25,26].

### 2.3. DNA Sequencing and Phylogenetic Analysis

For phylogenetic analysis a total 28 samples were selected. To this end, the amplicons obtained were sequenced using the Sanger method to obtain phylogenetic information and confirm the viral lineage and strain.

For phylogenetic analysis, the MEGA-X program [30] was employed. The evolutionary history was inferred using the maximum likelihood method and the Tamura–Nei model. The initial tree for the heuristic search was automatically obtained by applying the Neighbor-Join and BioNJ algorithms to a matrix of pairwise distances estimated using the Tamura–Nei model, and then selecting the topology with the highest log-likelihood value [30]).

## 3. Results

### 3.1. Prevalence of IBV in Vaccine and Non-Vaccine Samples

Out of the 100 batches assessed for IBV detection, 91% of the samples tested positive. The positive samples were further subtyped (except five samples that exhibited a low quality of genetic material were consequently categorized as untyped). In Table 2, the results of IBV detection in different Brazilian regions are presented.

From the positive samples, the subtyping revealed that 12 (14.63%) samples were characterized as GI-1 and 24 (29.26%) were classified as GI-11, 21 samples (25.6%) of them were categorized as positive for both GI-1 and GI-11, seven (8.53%) were classified as GI-11 and GI-23, three (3.65%) samples were identified as GI-1 and GI-23, three (3.65%) samples were classified as GI-1, GI-11, and GI-23, and none of the batches were classified as GI-23.

### 3.2. The S1 Glycoprotein Region Sequencing

**Figure 2:**
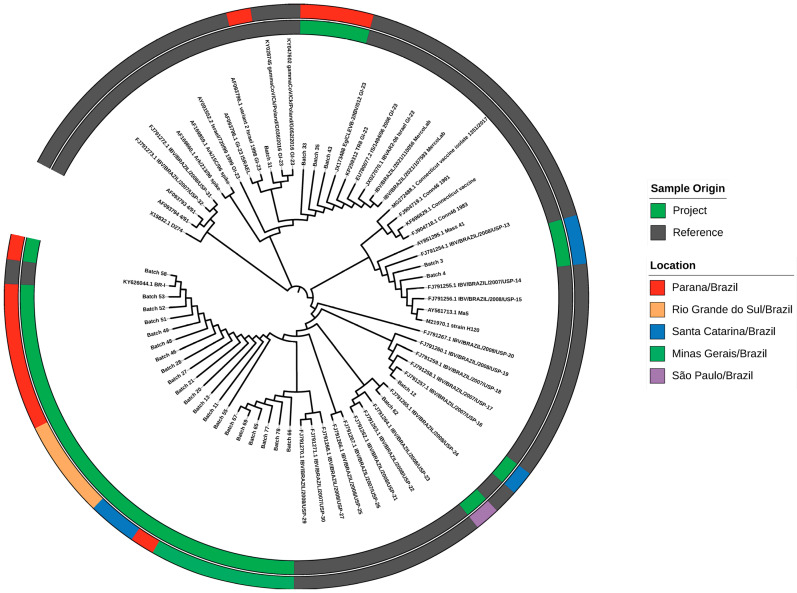
Phylogenetic analysis of partial S1 sequences of Brazilian and international IBV strains. The tree was generated by the maximum likelihood method and the Tamura–Nei model. The tree with the highest log likelihood (−2056.43) is shown. The percentage of trees in which the associated taxa cluster together is shown next to the branches. The initial tree for the heuristic search was automatically obtained by applying the Neighbor-Join and BioNJ algorithms to a matrix of pairwise distances estimated using the Tamura–Nei model, and then selecting the topology with the highest log-likelihood value. The tree is drawn to scale, with branch lengths measured in the number of substitutions per site.

Following the initial screening through RT-PCR, the sequencing of the S1 glycoprotein was carried out for potential differentiation between vaccine strains and field challenge strains, even to identify new strains in circulation within the national territory. These analyses were conducted on batches representing the production areas where strains that were potentially non-vaccine-related were detected (all regions were evaluated here, except for Ceará, as the IBV-positive samples, when typed, were positive for the Massachusetts vaccine strain). In this regard, 28 (32.9%) of the positive batches were found to contain strains related to field challenges. According to the phylogenetic relationship (GenBank OR972341-OR972368) presented in Figure 2, two batches (3 and 4) from the Santa Catarina State were grouped and classified as belonging to the GI-1 strain with the nucleotide sequences showing high similarity to the USP-13 samples (GenBank: FJ791254.1) and the H120 strain (GenBank: M21970.1).

The batches correspond to the states of Santa Catarina (11 and 13), Rio Grande do Sul (20, 21, 27, and 28), and Paraná (46, 48, 49, 51, 52, 53, 55, and 58), were clustered and categorized as members of the GI-11 strain. The nucleotide sequences of batches exhibited high similarity to the BR-I strain (GenBank: KY626044.1). The nucleotide sequence of batch 12 (from Santa Catarina state) showed high similarity to the USP-16 sample (GenBank: FJ791757.1).

The nucleotide sequence of batch 62 (from the São Paulo state), showed more than a 99.98% similarity to the USP-24 sample (GenBank: FJ91265.1). From the state of Minas Gerais (state that does not use IBV vaccines), the nucleotide sequence of six batches (65, 66, 67, 69, 77, and 78) from the southeast region exhibited high similarity with the samples USP-27 (GenBank: FJ791268.1), USP-29 (GenBank: FJ791270.1), and USP-30 (GenBank: FJ791271.1).

According to the phylogenetic relationship depicted in Figure 2, four batches (31, 33, 35, 43) were grouped within the GI-23 strain, all of them from the state of Paraná. The nucleotide sequence exhibited a similarity exceeding 99.99% with the IBV Israel variant-2 strain (GenBank: JX027070.1).

The comprehensive analysis of the S1 glycoprotein sequences demonstrates the diversity and distribution of IBV strains in different regions of Brazil. Identifying strains related to field challenges, particularly those not linked to vaccine strains, highlights the dynamic nature of IBV and the continuous evolution of the virus. The phylogenetic analysis, as depicted in Figure 2, provides a visual representation of the genetic relationships among Brazilian and international IBV strains.

### 3.3. Vaccine Persistence in Tracheal Samples

The Table 3 presents the state to which the batches belonged, the age of the birds, the vaccination program employed, and the typing results for batches where the persistence of the vaccine virus may have occurred.

In total, 67 samples exhibited viral vaccine persistence, regardless of the vaccination programs used by different companies. This study indicated vaccine persistence against IBV for up to 32 days after use, considering that the ages of the batches evaluated ranged from 16 to 32 days of life.

At the time of possible vaccine virus detection, the batches ranged in age from 16 to 32 days. Specifically, between days 16 and 21, vaccine persistence was observed in 14 batches; between days 22 and 27, it was noted in 38 batches; and from days 28 to 32, six batches displayed vaccine persistence.

## 4. Discussion

The first case of IBV in Brazil was linked to the GI-1 (mass serotype) occurred in the 1950s. Since then, the virus has evolved significantly, and new lineages have been identified over time [2]. The introduction of vaccines has been a crucial measure in mitigating productivity losses in both short-cycle and long-cycle poultry. Before 2016, vaccination against IBV was primarily conducted using the [12]. However, following respiratory outbreaks in birds and epidemiological identification of the genogroup GI-11 (BR strain), the Ministry of Agriculture, Livestock and Supplying (MAPA) approved the use of a novel vaccine for IBV prevention. This new vaccine utilized a strain homologous to the Brazilian variant, which had been previously described [31,32]. As this vaccine was integrated into Brazilian poultry batches, outbreaks decreased to some extent, contributing to better control over damage caused by this lineage [23]. In this context, the six states that comprised the epidemiological research in this study represent approximately 80% of the country’s chicken production [32].

In total, 90% of the birds received vaccines containing GI-1 lineage, either individually (30%) or in combination with vaccines containing GI-11 lineage (60%). Furthermore, 10% of the batches remained unvaccinated, which poses significant risks to the entire batch. In regions where no vaccination was implemented, a substantial proportion (70%) of samples tested positive for GI-11 lineage, highlighting the considerable sanitary risk associated with omitting vaccines from immunoprophylaxis strategies. Vaccination programs can vary substantially depending on factors such as country-specific regulations, regional biosecurity measures, and findings from molecular diagnostics. These elements are crucial when introducing new vaccines because regional production characteristics can differ significantly. Consequently, companies can make more informed decisions regarding their vaccination programs by considering these variables.

Two studies, also evaluating samples from 2021, mainly from the southern region, particularly from the state of Paraná, identified the presence of GI-23 lineage circulating in broilers. Since its introduction, there has been a rapid spread of this lineage among farms in the major producing regions of the country, with birds exhibiting severe clinical symptoms in the upper respiratory tract and renal lesions [24,25,33].

The diversity of vaccines for avian infectious bronchitis virus (IBV), including live-attenuated and killed types developed from local variant serotypes using conventional production strategies, supports customized vaccination programs across different regions or companies in Brazil. These programs effectively manage IBV infections by addressing unique regional challenges arising from heterogeneous strains and epidemiological conditions [21,34,35]. In the case of live vaccines, the aim is to simulate the natural infection process by the field virus, but in an attenuated manner, so that the virus replication induces an immune response without causing the complete disease [36].

In Paraná, out of 30 biological samples, 18 samples revealed a new IBV variant in Brazil. This discovery justifies the issues faced in that state, where many companies reported IBV outbreaks associated with high condemnations in slaughterhouses between 2020 and 2021. Outbreaks linked to this new variant may be related to the partial coverage of vaccination programs used during that period. As in the present study, the strains identified by Trevisol et al. (2023) [25], who conducted a phylogenetic analysis and grouped them with strains circulating in Poland and Israel, the strains identified by Ikuta et al. (2022) [24] were clustered with the parental GI23 strain from Israel (Variant-2), forming a distinct clade separated from all other IBV lineages [37,38].

The state of Santa Catarina was the only one that presented a challenge with lineage GI-1 (Mass strain), which may be related to vaccine application failures in hatcheries [37]. Outbreaks linked to this new variant may be related to partial coverage of the vaccination programs used during that period.

In Rio Grande do Sul, three biological samples tested positive for the GI-23 lineage, comprising 20% of the samples from this state. Despite employing a vaccination program that offers partial protection against this variant, it does not fully prevent infection or replication of the virus. This limitation underscores that no single live-attenuated vaccine provides comprehensive protection against all IBV strains, highlighting the need for tailored vaccination strategies to address specific regional challenges [35,37].

In the state of São Paulo, the vaccination program consisted only of the GI-1 (mass strain), providing partial protection for other serotypes, justifying the presence of GI-11 (BR-I strain). In Minas Gerais, the company did not use vaccines for IBV prevention, creating an opportunity for the infection and replication of field viruses. In this state, out of the 10 samples, seven were positive for IBV, with six of them being classified as GI-11 (BR-I strain), and a sample was related to the GI-1 (mass strain) vaccine virus. In this case, there may have been vaccine virus transmission from a neighboring company, thus, indicating the circulation of the GI-11 (BR-I strain) [39].

In Ceará, all subtyped samples were linked to vaccine strains. This is consistent with findings from numerous studies where vaccinated batches exhibit field strains upon molecular evaluation. Such outcomes underscore the importance of advanced diagnostic technologies in identifying these dynamics [24].

One of the primary concerns surrounding the ongoing use of live vaccines is their potential to alter pathogenic populations in the environment through mechanisms such as genomic recombination, reversion to virulence, or strain replacement facilitation. This concern becomes particularly pronounced when a new vaccine strain is introduced into regions where the corresponding pathogenic variant has not been previously identified, as the vaccine strain may evolve unpredictably once introduced. This unpredictability creates additional challenges for disease control strategies by complicating epidemiological surveillance and management efforts [36,40,41].

It is not clear how poultry can respond to the combination of two or more IBV vaccines, which could be a limiting factor in protecting the birds against challenges [11]. The high percentage of vaccine viruses found in the birds may be related to the high excretion of the virus in the facilities, leading to viral transmission. This also raises the hypothesis of a high rate of virus persistence in the host, which is similar to what has been demonstrated [38]. The concentration of vaccine viruses is highest after vaccine administration on the first day of life, and it decreases as the birds’ immunity is established [37].

In general, the results emphasize the need for continued surveillance and monitoring of IBV in poultry batches in Brazil. The genetic information obtained in this study can serve as a foundation for the development of more targeted and efficient vaccines. Tailoring vaccines to specific lineages and variants can enhance their efficacy in mitigating IBV outbreaks.

## 5. Conclusions

Of the total batches evaluated, 91% (91/100) presented positive results for IBV, demonstrating the simultaneous or isolated circulation of GI-1, GI-11, 21, and/or GI-23 in Brazil. In addition, this study indicated vaccine persistence against IBV for up to 32 days after use, considering that the vaccines were administered on the first day of life of the animals and that the ages of the batches evaluated ranged from 16 to 32 days of life.

The data demonstrate a widespread occurrence of infectious bronchitis virus (IBV) in Brazil’s broiler flocks, with prolonged viral persistence observed in vaccinated birds and the presence of wild-type viruses in unvaccinated birds or those vaccinated against specific strains.

## Figures and Tables

**Table 1:** Demonstrate the different vaccination programs in Brazilian states. Each region utilizes programs based on its local challenges, which may vary between the use of one or two strains in the same immunoprevention program, where the day and method of application are the same for all companies.

State	Vaccination Program	Vaccination Date	Location and Vaccination Route
Santa Catarina	GI-1 + GI-11	First day of life	Spray in the hatchery
Rio Grande do Sul	GI-1 + GI-11		
Paraná	GI-1 + GI-11		
Minas Gerais	Unvaccinated birds for IBV		
São Paulo	GI-1		
Ceará	GI-1		

**Table 2:** Prevalence of IBV in the different states that originated from the samples, showing the total number of batches that exhibited viral persistence, whether with the presence of one or more IBV strains. Additionally, it is possible to verify the age of the evaluated animals.

State	Age of Collection (Days Old)	Total Samples	Number of Positive Samples—IBV	Suspected IBV Strains Identified Through RT-PCR
Rio Grande do Sul	16–26	15	14	(07) GI-11 (01) GI-11, GI-23 (05) GI-1, GI-11 (01) GI-1, GI-11, GI-23
Santa Catarina	17–25	15	14	(02) GI-1 (02) GI-11 (10) GI-1, GI-11
Paraná	20–32	30	25	(11) GI-11 (03) GI-1, GI-11 (06) GI-11, GI-23 (02) GI-1, GI-11, GI-23 (03) GI-1, GI-23
São Paulo	21–27	10	5	(01) Untyped (01) GI-11 (02) GI-1, GI-11 (01) GI-1
Minas Gerais	13–20	10	10	(07) Untyped (03) GI-11
Ceará	24–28	20	14	(04) Untyped (01) GI-1, GI-11 (09) GI-1

**Table 3:** Information on the state affiliation of the batches, the age of the birds, the employed vaccination program, and the IBV genotype results for batches where the persistence of the vaccine virus is potentially observed.

State	Batch Number	Age (Days)	Vaccine	IBV Genotype
Santa Catarina	1	17	Infectious bronchitis—GI-1 (live) + GI-11 (live)	GI-1 and GI-11
	2	17		GI-1 and GI-11
	3	19		GI-1
	4	25		GI-1
	5	25		GI-1 and GI-11
	6	20		GI-1 and GI-11
	7	18		GI-1 and GI-11
	8	18		GI-1 and GI-11
	9	17		GI-1 and GI-11
	10	21		GI-1 and GI-11
	11	22		GI-11
	13	19		GI-11
	14	18		GI-1 and GI-11
	15	17		GI-1 and GI-11
Rio Grande do Sul	16	24	Infectious bronchitis—GI-1 (live) + GI-11 (live)	GI-11 and GI-23
	17	23		GI-11
	18	26		GI-1 and GI-11
	19	27		GI-1, GI-11, and GI-23
	20	21		GI-11
	21	20/21		GI-11
	22	24		GI-1 and GI-11
	23	24		GI-1 and GI-11
	24	16		GI-1 and GI-11
	25	22		GI-11
	26	22		GI-1 and GI-11
	27	21		GI-11
	28	21		GI-11
	29	22		GI-11
Paraná	32	32	Infectious bronchitis—GI-1 (live) + GI-11 (live) Infectious bronchitis—GI-1 (live) + GI-11 (live)	GI-1 and GI-23
	34	25		GI-1 and GI-23
	36	21		GI-11 and GI-23
	37	24		GI-11 and GI-23
	38	24		GI-11 and GI-23
	39	22		GI-1, GI-11, and GI-23
	41	31		GI-1, GI-11, and GI-23
	42	28		GI-1 and GI-23
	44	32		GI-11 and GI-23
	45	27		GI-11 and GI-23
	46	22		GI-11
	47	23		GI-11
	48	23		GI-11
	49	25		GI-11
	50	25		GI-1 and GI-11
	51	25		GI-11
	52	25		GI-11
	53	25		GI-11
	54	20		GI-1 and GI-11
	55	24		GI-11
	56	20		GI-11 and GI-23
	57	25		GI-11
	58	25		GI-11
	59	24		GI-1, GI-11
	60	24		GI-11
São Paulo	62	26	Infectious bronchitis—GI-1 (live)	GI-11
	71	23		GI-1 and GI-11
	72	27		GI-1 and GI-11
	73	23		GI-1
Ceará	81	24	Infectious bronchitis—GI-1 (live)	GI-1
	84	24		GI-1 and GI-11
	85	24		GI-1
	87	24		GI-1
	89	24		GI-1
	91	24		GI-1
	92	24		GI-1
	94	24		GI-1
	95	24		GI-1
	98	24		GI-1

## Data Availability

The original contributions presented in this study are included in the article. Further inquiries can be directed to the corresponding author.

## Abbreviations

The following abbreviations are used in this manuscript:

MDPI	Multidisciplinary Digital Publishing Institute
DOAJ	Directory of Open Access Journals
TLA	Three-letter acronym
LD	Linear dichroism
